# Epidemiological parameters of COVID-19 and its implication for infectivity among patients in China, 1 January to 11 February 2020

**DOI:** 10.2807/1560-7917.ES.2020.25.40.2000250

**Published:** 2020-10-08

**Authors:** Qing-Bin Lu, Yong Zhang, Ming-Jin Liu, Hai-Yang Zhang, Neda Jalali, An-Ran Zhang, Jia-Chen Li, Han Zhao, Qian-Qian Song, Tian-Shuo Zhao, Jing Zhao, Han-Yu Liu, Juan Du, Ai-Ying Teng, Zi-Wei Zhou, Shi-Xia Zhou, Tian-Le Che, Tao Wang, Tong Yang, Xiu-Gang Guan, Xue-Fang Peng, Yu-Na Wang, Yuan-Yuan Zhang, Shou-Ming Lv, Bao-Cheng Liu, Wen-Qiang Shi, Xiao-Ai Zhang, Xiao-Gang Duan, Wei Liu, Yang Yang, Li-Qun Fang

**Affiliations:** 1 Department of Laboratorial Science and Technology, School of Public Health, Peking University, Beijing, China; 2 These authors contributed equally to this manuscript; 3 School of Mathematical Sciences, Beijing Normal University, Beijing, China; 4 Department of Biostatistics, College of Public Health and Health Professions, and Emerging Pathogens Institute, University of Florida, Gainesville, United States; 5 State Key Laboratory of Pathogen and Biosecurity, Beijing Institute of Microbiology and Epidemiology, Beijing, China; 6 School of Statistics, Beijing Normal University, Beijing, China; 7 These senior authors contributed equally to this manuscript

**Keywords:** coronavirus disease 2019, SARS-CoV-2, incubation period, generation interval, serial interval, China

## Abstract

**Background:**

The natural history of disease in patients infected with severe acute respiratory syndrome coronavirus 2 (SARS-CoV-2) remained obscure during the early pandemic.

**Aim:**

Our objective was to estimate epidemiological parameters of coronavirus disease (COVID-19) and assess the relative infectivity of the incubation period.

**Methods:**

We estimated the distributions of four epidemiological parameters of SARS-CoV-2 transmission using a large database of COVID-19 cases and potential transmission pairs of cases, and assessed their heterogeneity by demographics, epidemic phase and geographical region. We further calculated the time of peak infectivity and quantified the proportion of secondary infections during the incubation period.

**Results:**

The median incubation period was 7.2 (95% confidence interval (CI): 6.9‒7.5) days. The median serial and generation intervals were similar, 4.7 (95% CI: 4.2‒5.3) and 4.6 (95% CI: 4.2‒5.1) days, respectively. Paediatric cases < 18 years had a longer incubation period than adult age groups (p = 0.007). The median incubation period increased from 4.4 days before 25 January to 11.5 days after 31 January (p < 0.001), whereas the median serial (generation) interval contracted from 5.9 (4.8) days before 25 January to 3.4 (3.7) days after. The median time from symptom onset to discharge was also shortened from 18.3 before 22 January to 14.1 days after. Peak infectivity occurred 1 day before symptom onset on average, and the incubation period accounted for 70% of transmission.

**Conclusion:**

The high infectivity during the incubation period led to short generation and serial intervals, necessitating aggressive control measures such as early case finding and quarantine of close contacts.

## Introduction

A novel coronavirus, severe acute respiratory syndrome coronavirus 2 (SARS-CoV-2), emerged in Wuhan, China near the end of 2019 and has caused an unprecedented outbreak of pneumonia, named coronavirus disease (COVID-19), in China and many parts of the world [1,2]. By 25 September 2020, a total of 85,337 confirmed and probable cases, including 4,634 deaths, have been reported from 31 provinces of the mainland of China [3]. The efficient transmission and clinical severity of SARS-CoV-2 have raised challenges in the containment of this epidemic. While scientists are developing antiviral drugs and vaccines targeting this virus, non-pharmaceutical control measures such as early diagnosis, contact tracing followed by quarantine, and restriction of human movement have been used to slow down the spread the disease and minimise its impact.

Success or failure of these measures depend on the epidemiological parameters governing the natural history of disease and transmissibility of SARS-CoV-2, e.g. basic reproductive number (R_0_), distribution of incubation period and distribution of generation interval. Incubation period is the time lag from infection to symptom onset of an infected person [4], which is critical for the detection of potential cases. For example, close contacts of cases are often observed or quarantined for a period long enough to cover at least the 95th percentile of the incubation period distribution [5]. Generation interval is the time lag from a primary infection to a secondary infection caused by the primary infection. Both incubation period and generation interval, in addition to R_0_, are key parameters for assessing transmission dynamics, predicting epidemic trends and evaluating intervention effectiveness [6‒10]. A long average incubation period coupled with a short average generation interval indicates potential transmission of the pathogen during the incubation period and thus implies that it is difficult to contain the epidemic as an infectious person in their incubation period is hard to identify and is likely to be moving around. Serial interval is the time interval between symptom onset in a primary case and a secondary case, which is considered a reasonable approximation of the generation interval and is more practical to observe [11].

Both R_0_ and the distribution of the incubation period of COVID-19 have been estimated using epidemiological investigation data in the early phase of the epidemic [1,12‒17]. However, we lack a full account of potential spatial and temporal heterogeneity in the observed distribution of the incubation period, which is important to tailor intervention programmes. Spatiotemporal heterogeneity may result from possible dependence of the incubation period distribution on disease severity coupled with surveillance bias, e.g. severe cases were more likely to be ascertained than mild cases during the early outbreak compared with the later phase as well as in heavily affected regions compared with where there were only a handful of cases. Much less is known about the distribution of the serial or generation intervals, mostly because there is no centralised publicly accessible database containing exposure information for identified cases.

Here we collected and reviewed all publicly accessible and de-identified COVID-19 cases and clusters in mainland China available on 11 February 2020. Most data were released to the public by local Chinese public health authorities after thorough epidemiological investigations of newly diagnosed and confirmed cases, with the hope that close contacts of these cases not covered by the investigations may seek help or healthcare. The wide availability of such data made it possible to examine potential demographic and spatiotemporal heterogeneity in the natural history of disease. With this newly built centralised database, we estimated the distributions of the incubation period, serial interval and generation interval, accounting for uncertainty in the exposure history of patients. Using these distributions, we estimated the proportion of infections that occurred during the incubation period. In addition, we summarised the distributional features for the time from symptom onset to hospital discharge which constitutes an important part of the infectious period of COVID-19 patients [18].

## Methods

### Data sources

We collected and reviewed all exposure and symptom onset data on de-identified, laboratory-confirmed COVID-19 cases in mainland China as available on 11 February 2020. We searched the Internet using keywords in the form of (‘coronavirus’ OR ‘pneumonia’) AND (province or city names), all in Chinese. Relevant data were extracted from both news media and formal announcements by district, municipal and provincial branches of the Chinese Center for Disease Control and Prevention (CDC) and Health Commissions, as well as literature, mostly in Chinese. Basic demographic characteristics (age, sex and location), probable starting and stopping dates of exposure, and dates of symptom onset, diagnosis, hospitalisation and discharge, laboratory diagnosis status and associated epidemiological cluster were retrieved for each identified COVID-19 case and organised as an electronic master database. Cases without laboratory confirmation were excluded from the master database. Laboratory-confirmed case for whom both onset dates and probable exposure period were available were included to estimate the distribution of the incubation period. Exposure was determined by (i) recent residence in or travel to Wuhan, (ii) existence of an epidemiological link with another potential source case who had either an earlier symptom onset or recent residence in or travel to Wuhan, or (iii) recent contact with confirmed cases from Wuhan. Cases with information on age, sex or location were further used for subgroup-specific estimation. 

**Figure 1:**
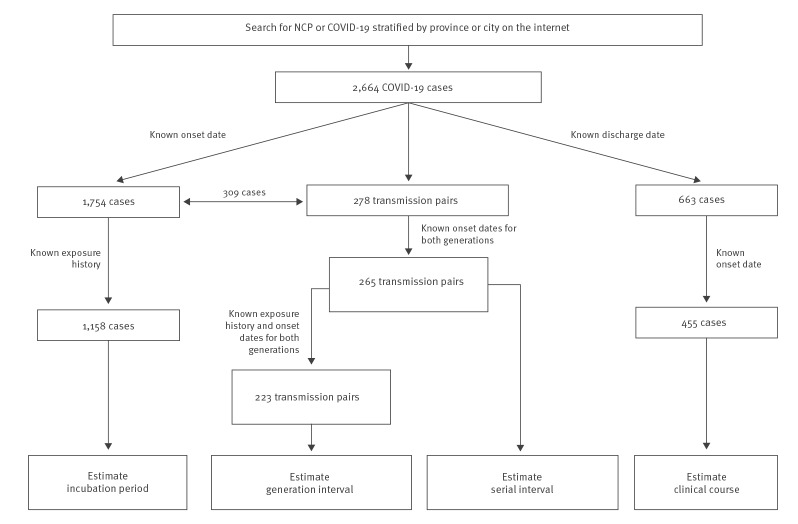
Flowchart of COVID-19 patients and transmission pairs screened for estimating distributions of the incubation period, serial interval, generation interval and time from symptom onset to discharge, China, 1 January–11 February 2020 (n = 2,664)

COVID-19: coronavirus disease; NCP: novel coronavirus pneumonia.

To estimate distributions of the serial and generation intervals, we identified potential transmission pairs, i.e. a pair formed by a primary case and one of their secondary cases, from clusters of epidemiologically linked cases in the master database. In such a pair, symptom onset dates for both cases were known, but only the primary case, not the secondary case, had lived in or travelled to Wuhan or had a clear evidence of contact with an earlier confirmed case. We did not solely use the symptom onset dates to determine a transmission pair, given the long and variable incubation period suggested in literature [1,13,16]. When the starting date of exposure was missing, we assumed that it was no more than 3 weeks before the symptom onset date. For a secondary case, the starting date of exposure was further assumed to be no earlier than that of the primary case. When the stopping date of exposure was missing, we set it to 1 day before symptom onset. Lastly, all cases with available symptom onset and discharge dates were included to estimate the distribution of time from symptom onset to discharge. A flowchart showing exact numbers at each screening stage is given in Figure 1.

The data sources and the derived database were cross-examined by multiple research team members (BIME, PU and UF) multiple times. The majority of our data was obtained from official websites of public health authorities (63%), websites of credible news media such as sina.com (27%), and the official account of a mainstream domestic social media provider (WeChat, 8%). In addition, 227 (81.7%) transmission pairs we identified were reported by more than one website (Supplementary Figure S1). The symptom onset dates of cases in our database showed a similar temporal pattern to that of officially reported confirmed cases in China (obtained from the website of the National Health Commission), and similarity was also seen for cases reported in Guangdong and Henan provinces (Supplementary Figure S2), suggesting our data constituted a representative subset of the overall epidemic.

### Statistical analyses

All distributions were fitted by parametric models accounting for interval-censoring, and the maximum likelihood approach was used [5,19‒21]. For the incubation period, serial interval and the time from symptom onset to discharge, we considered log-normal, gamma, log-logistic and Weibull distributions. For the generation interval, we considered Weibull and log-logistic models. The log-logistic distribution can accommodate unimodal hazard functions, which is likely to meet the empirical experience of how infectivity changes over time, whereas Weibull and gamma provide only monotone hazard functions [22]. The best fitted model was determined by the Akaike's information criterion (AIC). Standard errors, 95% confidence intervals (CI) and p values were obtained with parametric bootstrapping.

Specifically, for each case *i*, let *T_i_^E^*, *T_i_^S^* and *T_i_^D^* be the exposure (infection), symptom onset and hospital discharge dates, respectively. The incubation period is then *V_i_^Inc^* = *T_i_^S^*−*T_i_^E^*, and the time from symptom onset to discharge is *V_i_^Dis^* = *T_i_^D^*−*T_i_^S^*. The exact exposure date is usually not directly observed but rather bounded by an interval, i.e. *L_i_* ≤ *T_i_^E^* ≤ *U_i_*, and the incubation interval is thus bounded by *T_i_^S^*–*U_i_* ≤ *V_i_^Inc^* ≤ *T_i_^S^*−*L_i_*. Occasionally, we have *T_i_^S^* = *U_i_*, for which we let the lower bound of *V_i_^Inc^* be 0.5. Suppose patient *i* and *j* form a transmission pair with *i* as the primary case and *j* as the secondary case. The generation and serial intervals are *V_i_^GI^* = *T_i_^E^*−*T_j_^E^* and *V_i_^SI^* = *T_i_^S^*−*T_j_^S^*, respectively. 

**Figure 2:**
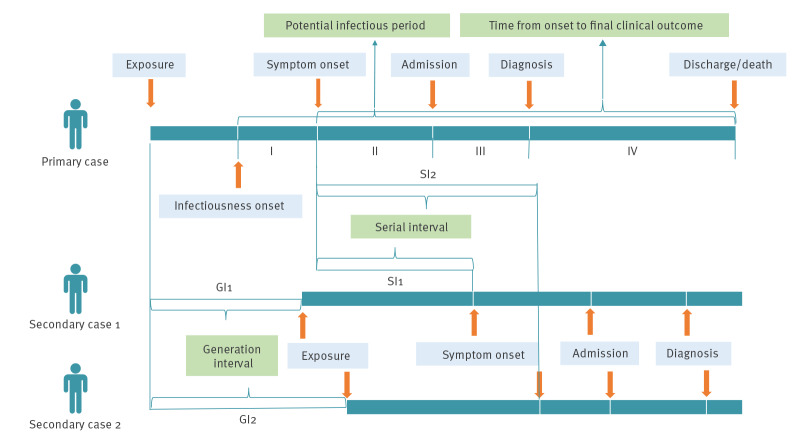
Schematic of the incubation period, serial interval, generation interval and time from symptom onset to discharge, COVID-19 patients, China

GI: generation interval; SI: serial interval.

The conceptual structure and relationships of all the intervals studied here are shown in a schematic plot (Figure 2). 

We first estimated the distributions of the incubation period and serial interval using the package ‘fitdistrplus’ of the statistical software R, accounting for interval censoring of the incubation period. As the observed serial intervals could be negative values, we shifted all observed values to be positive by adding 10 days and then shifted the estimates back to the original scale. The distribution of the incubation period was then assumed known and fixed in the subsequent estimation for the generation interval. This is a reasonable assumption as there were many more available individual patients than available transmission pairs. The likelihood used for estimating the distribution of the generation interval was 

$$\prod_{i}\sum_{T_{i}^{E}=L_{i}}^{U_{i}}f_{Inc}(T_{i}^{S}-T_{i}^{E})\sum_{T_{j}^{E}=max(L_{j},T_{i}^{E})}^{U_{j}}f_{Inc}(T_{j}^{S}-T_{j}^{E})f_{GI}(T_{j}^{E}-T_{i}^{E})$$,

where *f_Inc_*() and *f_GI_*() are the probability density functions for the incubation period and the generation interval, respectively. The time of peak infectivity was calculated as the mode of the hazard function, *f_GI_* (*t*)/[1−*F_GI_* (*t*)] , where *F_GI_* (*t*) is the cumulative density function of the generation interval. For example, for a log-logistic distribution with shape *α* and rate *γ*, the mode is given by 1/*γ*(*α−1)^1/α^*. The proportion of secondary transmissions that occurred during the incubation period of primary cases was calculated as $\int_{0}^{\infty }f_{Inc}(t)F_{GI}(t)dt$. This is the probability that the infection of the secondary case occurred during the incubation period of the primary case, on the condition that a transmission occurred between the two. It cannot be interpreted as the probability of infection of a susceptible person by an infectious close contact during their incubation period. Confidence intervals of all quantities were obtained using parametric boostrapping. All analyses were conducted using R version 3.6.0 (R Development Core Team, Vienna, Austria) [23].

### Ethical statement

This study was approved by the institutional review board of the Beijing Institute of Microbiology and Epidemiology (Beijing, China, 20J009). All data were collected from publicly available sources. Data were de-identified, and informed consent was waived.

## Results

Our search yielded 1,754 confirmed COVID-19 cases with known symptom onset dates and 278 transmission pairs (Figure 1). After excluding cases and transmission pairs with no or conflicting exposure history, we had 1,158 cases for the analysis of the incubation period, and 265 and 223 transmission pairs for the estimation of, respectively, the serial and generation interval. We did a separate search by adding ‘discharge’ or ‘recover’ to the keywords and found 663 patients with discharge dates, among whom 455 also had symptom onset dates and could therefore be used towards the analysis of the time to discharge. Baseline demographic data of the patients used for inference with regard to incubation period, serial interval (primary cases only) and time to discharge are summarised in Supplementary Table S1. The pattern of the temporal distribution of cases in our database was similar to that of the reported overall number of cases in China as a whole and in two Chinese provinces, indicating that our data were representative for the overall epidemic in the country (Supplementary Figure S2).

The three non-exclusive groups of patients, 1,158 cases for estimating the incubation period, 265 index cases of the transmission pairs for estimating the serial interval, and 455 patients with symptom onset and discharge dates, are here referred to as general patients, transmitters and discharged patients, respectively. Discharged patients were younger (median = 39 years; interquartile range (IQR): 31‒50; p < 0.001), while transmitters were older (median = 46 years; IQR: 36‒57; p = 0.027) than the general patients (median = 43 years; IQR: 33‒56) (Supplementary Table S1). Transmitters were more frequently male and residents in the northern provinces, compared with the other two groups (both p < 0.001). Northern provinces include Gansu, Shaanxi, Henan, Shandong and those to the north of these provinces, and southern provinces are those to the south of these provinces. The majority of the discharged patients in our data were from southern provinces (including Hubei), but this does not necessarily imply that recovery rate is higher in the south.

**Figure 3:**
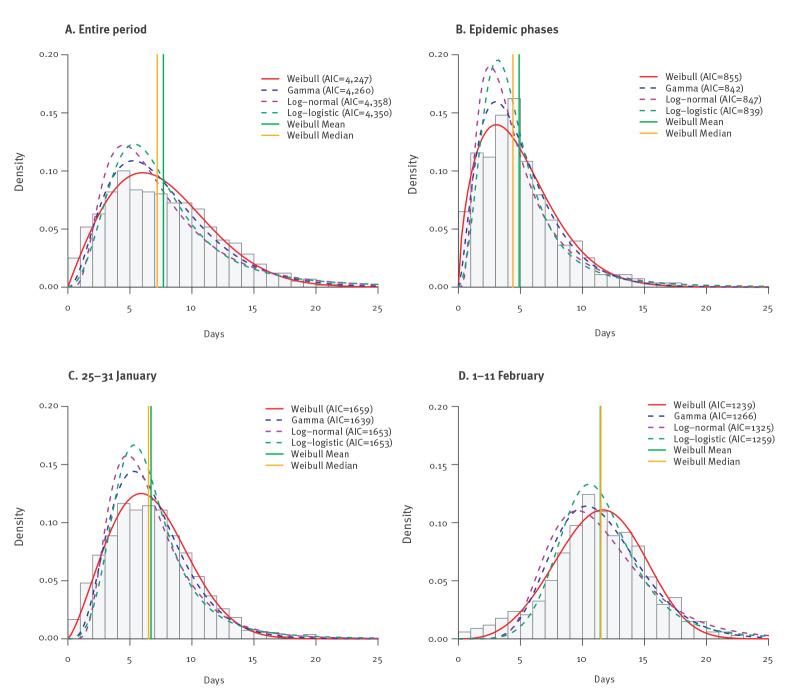
Estimated distributions of the incubation period based on public data on COVID-19 cases, China, 1 January–11 February 2020 (n = 1,158)

AIC: Akaike's information criterion; COVID-19: coronavirus disease.

The best fitted parametric model for the incubation period based on all 1,158 patients was the Weibull distribution, although the best model could differ for different subgroups of the patients (Figure 3). We present the estimates based on the Weibull model as the primary results. The median incubation period was estimated to be 7.2 days (95% CI: 6.9–7.5), but its 95th and 99th percentiles could be as long as 15.1 and 18.7 days (Table 1). The probability of the incubation period being longer than 20 days was ca 0.6%. The median incubation period increased significantly from 4.4 days (95% CI: 4.0–4.9) before 25 January 2020 to 6.5 days (95% CI: 6.1–6.8) during 25‒31 January 2020, and further to 11.5 days (95% CI: 11.1–12.0) after 31 January 2020 (p < 0.001). The 95th and 99th percentiles also increased significantly, from 14 days to 19.2 days for the 99th percentile. Children (< 18 years) had slightly longer incubation period, a median of 8.8 days (95% CI: 7.3–10.5), compared with 6.9‒7.2 days among adults (p = 0.007, comparing children to all adults). No difference was found between female and male patients or between people living in the south and those living in the north (Table 1).

Epidemic phases are defined by date of symptom onset. Parametric models fitted are Weibull (red solid), gamma (blue dashed), log-normal (purple dashed) and log-logistic (green dashed). Density of observed distribution (using middle point of exposure interval as exposure time) is shown as the histogram. Vertical lines indicate mean (green) and median (orange) of the Weibull distribution.

**Table 1:** Estimates for the incubation period based on the Weibull distribution for the COVID-19 epidemic, by age group, sex, epidemic phase and location, China, 1 January–11 February 2020 (n = 1,158)

Incubation period (days)	Number of cases	Median		95th percentile		99th percentile	
		Estimate	95% CI^a^	Estimate	95% CI^a^	Estimate	95% CI^a^
Age^b ^(years)							
< 18	61	8.8	7.3–10.5	18.0	14.8–20.8	22.3	16.8–25.4
18‒44	500	7.1	6.7–7.6	14.5	13.6–15.3	17.8	16.1–19.2
45‒59	334	6.9	6.4–7.4	14.3	13.2–15.4	17.8	15.7–19.3
≥ 60	193	7.2	6.4–8.0	15.6	14.0–17.2	19.6	16.6–22.2
Sex^b^							
Female	568	7.3	6.8–7.7	15.2	14.2–16.1	18.9	17.2–20.4
Male	587	7.1	6.7–7.5	14.9	14.0–15.8	18.5	16.9–20.0
Location							
South	640	7.0	6.6–7.4	14.8	14.0–15.7	18.5	17.0–20.0
North	518	7.4	6.9–7.9	15.3	14.4–16.3	19.0	17.2–20.4
Phase							
Before 25 Jan 2020	278	4.4	4.0–4.9	10.8	9.6–11.8	14.0	11.9–15.8
25‒31 Jan 2020	543	6.5	6.1–6.8	12.2	11.5–12.8	14.7	13.5–15.7
After 31 Jan 2020	337	11.5	11.1–12.0	17.1	16.3–17.8	19.2	18.1–20.2
Overall	1,158	7.2	6.9–7.5	15.1	14.4–15.7	18.7	17.6–19.8

CI: confidence interval; COVID-19: coronavirus disease.

^a^ 95% CI was calculated using 1,000 parametric bootstrap samples.

^b ^Age and sex data were not available for 70 and three cases, respectively.

**Figure 4:**
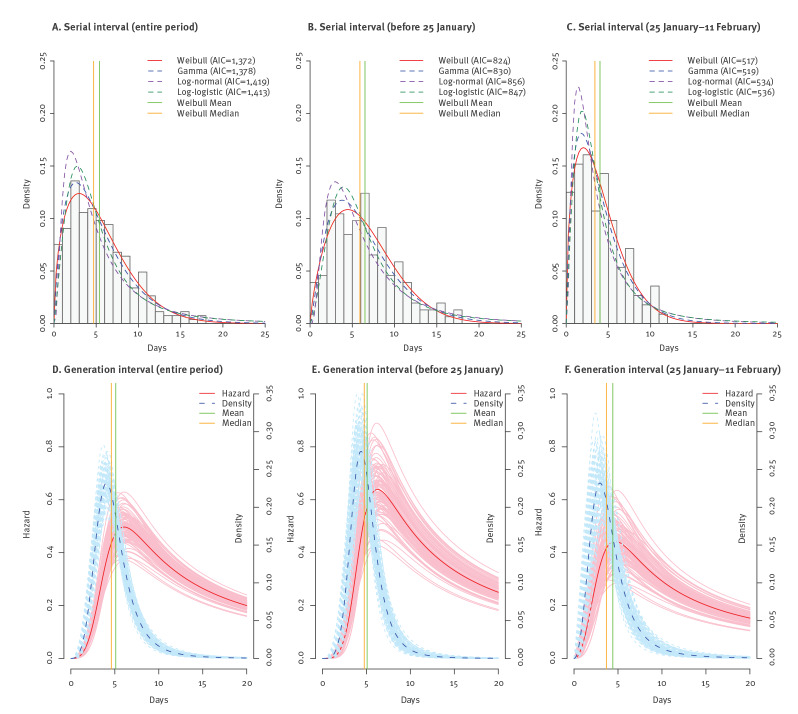
Parametric estimates of the distributions of serial and generation interval based on public data on COVID-19 cases, China, 1 January–11 February 2020 (n = 265 and 223, respectively)

AIC: Akaike's information criterion; COVID-19: coronavirus disease.

The Weibull distribution provided the best fit to the serial interval, although gamma distribution also fitted the data well (Figure 4A‒C). The median serial interval was 4.7 days (95% CI: 4.2–5.3). The 5th and 95th percentiles, estimated as 0.9 (95% CI: 0.6–1.2) and 12.4 (95% CI: 11.0–13.7) days, respectively, suggested a wide range of variation (Table 2). The serial median interval shrank substantially from 5.9 days (95% CI: 5.1–6.7) before 25 January to 3.4 days (95% CI: 2.9–4.1) after (p < 0.001), and so did the 5th and 95th percentiles. No difference was seen between northern and southern patients. The pattern of the generation intervals very much resembled that of the serial interval, and the log-logistic distribution also best fitted the generation interval. The incubation period distribution used for estimating the generation interval under each setting in Table 2 corresponds to the one listed in Table 1 under the same setting, except that the two phases after 25 January were combined into one owing to data availability. The overall median generation interval was 4.6 days (95% CI: 4.2–5.1) and the median was somewhat longer before 25 January than after, 4.8 (95% CI: 4.3–5.3) vs 3.7 (95% CI: 3.0–4.5) days. However, unlike the serial interval, the generation interval during the later epidemic phase was more skewed to the right, with a slightly longer 95th percentile than during the early phase (Table 2). No geographical heterogeneity was seen in the distribution of the generation interval. Nor did we find significant differences in serial or generation intervals between age group (45 years vs ≥ 45 years) or sex of the primary or secondary case (data not shown).

Epidemic phases are defined by date of symptom onsets before 25 January 2020 (B, E) and during 25 January 2020–11 February 2020 (C, F). Observed distributions are shown as histograms, parametrically fitted density functions are solid (best fitting) and dashed lines (other three estimates). For serial intervals, parametric models fitted are Weibull (red solid), gamma (blue dashed), log-normal (purple dashed) and log-logistic (green dashed). Density of observed serial intervals is shown as a histogram. Vertical lines indicate mean (green) and median (orange) of the Weibull distribution. For the generation interval, the estimated hazard function (red solid) and probability density function (blue dashed) shown are based on the log-logistic model, and parametric bootstrap samples are shown in light red for the hazard and in light blue for the density. Mean and median generation interval are indicated by the green and orange vertical lines.

**Table 2:** Estimates for the serial interval, generation interval and time from symptom onset to discharge for the COVID-19 epidemic based on the log-logistic model, by epidemic phase and location, China, 1 January–11 February 2020 (n = 265, 223 and 455, respectively)

Epidemiological parameter	Number of cases	5th percentile		Median		95th percentile	
		Estimate	95% CI^a^	Estimate	95% CI^a^	Estimate	95% CI^a^
Serial interval^b ^in days							
Location							
South	125	0.7	0.4–1.2	4.6	3.8–5.5	13.3	10.8–15.5
North	140	1.0	0.7–1.6	4.9	4.2–5.5	11.6	9.9–13.0
Phase							
Before 25 Jan 2020	153	1.3	0.9–2.0	5.9	5.1–6.7	13.7	11.9–15.2
25 Jan ‒11 Feb 2020	112	0.6	0.3–1.0	3.4	2.9–4.1	9.3	7.6–10.9
Overall^c^	265	0.9	0.6–1.2	4.7	4.2–5.3	12.4	11.0–13.7
Generation interval^b^ in days							
Location							
South	99	2.2	1.6–2.8	4.6	4.0–5.3	9.6	7.7–12.5
North	124	2.2	1.7–2.8	4.6	4.1–5.2	9.6	8.0–12.0
Phase							
Before 25 Jan 2020	134	2.6	2.1–3.2	4.8	4.3–5.3	8.6	7.3–10.4
25 Jan‒11 Feb 2020^d^	89	1.4	0.9–2.0	3.7	3.0–4.5	9.6	7.3–13.6
Overall^c^	223	2.2	1.8–2.6	4.6	4.2–5.1	9.6	8.3–11.3
Time from symptom onset to discharge in days^e^							
Age (years)							
< 18	16	9.1	7.4–11.9	14.0	12.2–16.0	21.7	16.4–27.1
18‒44	228	10.2	9.4–11.2	15.7	15.2–16.3	24.2	22.2–26.1
45‒59	117	12.0	10.9–13.3	17.4	16.7–18.2	25.2	22.9–28.0
≥ 60	50	11.1	9.4–13.6	18.3	16.7–20.2	30.1	24.7–36.4
Sex							
Female	205	10.7	9.7–11.7	16.7	16.0–17.3	25.9	23.6–28.4
Male	225	10.3	9.4–11.3	16.0	15.4–16.6	25.0	22.8–27.3
Location							
South	367	10.5	9.7–11.2	16.3	15.8–16.9	25.5	23.8–27.4
North	88	9.9	8.6–11.4	15.6	14.6–16.6	24.5	21.2–28.1
Hubei alone	84	11.0	9.7–12.7	17.3	16.2–18.5	27.3	24.0–31.8
Phase							
Before 22 Jan 2020	244	12.6	11.7–13.6	18.3	17.7–18.9	26.7	24.9–28.8
22 Jan‒11 Feb 2020	211	9.5	8.8–10.3	14.1	13.6–14.6	21.0	19.4–22.8
Overall^c^	455	10.4	9.8–11.0	16.2	15.7–16.7	25.3	23.9–27.0

CI: confidence interval; COVID-19: coronavirus disease.

^a^ 95% CI were calculated using 1,000 parametric bootstrap samples.

**^b^** Serial and generation intervals were estimated according to date of symptom onset and locations of primary cases.

^c ^The overall number of cases corresponds to the actual number used for each analysis.

^d^ The incubation period distribution used to estimate the generation interval for the phase 25 Jan‒11 Feb 2020 is Weibull (shape = 2.31, scale = 9.64) with a median of 8.2 days.

^e^ Estimates for the time from onset to discharge are further stratified by age group and sex.

Based on the estimates of the incubation period and generation interval, we further assessed the peak infectivity time and the proportion of secondary transmissions during the incubation period (Table 3). The hazard function of the generation interval began to increase sharply ca 2 days after exposure, peaked around 6.1 days (95% CI: 5.5–6.7) and descended at a lower rate afterwards (Figure 4D, Table 3). The infectivity peaked sooner during the later epidemic phase, ca 4.7 days (95% CI: 3.6–5.8) after exposure, than during the earlier phase with 6.3 days (95% CI: 5.7–6.9) (Figure 4E-F, Table 3). In addition, the overall infectivity level was lower during the later phase. Notably, these peak times occurred before the average time of symptom onset, 7.2 days after exposure. No difference was found between northern and southern China. Overall, ca 70% of secondary transmissions (95% CI: 66‒74) occurred during the incubation period. This proportion was much higher during the later epidemic phase, 83% (95% CI: 76‒88) compared with the earlier phase with 45% (95% CI: 39‒50).

**Table 3:** Estimates for the peak time of infectivity after exposure and the probability of secondary transmission during the incubation period rather than after symptom onset of a primary case, by epidemic phase and location, COVID-19 epidemic, China, 1 January–11 February 2020 (n = 223)

Infectivity after exposure	Peak infectivity time after exposure		Probability of transmission during incubation	
	Estimate (days)	95% CI^a^	Estimate (%)	95% CI^a^
Location				
South	6.0	5.1–7.0	69	62–75
North	6.1	5.3–6.9	72	66–77
Phase				
Before 25 Jan 2020	6.3	5.7–6.9	45	39–50
25 Jan‒11 Feb 2020	4.7	3.6–5.8	83	76–88
Overall	6.1	5.5–6.7	70	66–74

CI: confidence interval; COVID-19: coronavirus disease.

^a^ 95% CI were calculated using 1,000 parametric bootstrap samples.

The sample median of observed times from symptom onset to hospital discharge was 16 days (IQR: 13‒18). According to the AIC criteria, the best-fitting distribution was log-logistic (Supplementary Figure S3), yielding a median of 16.2 days (95% CI: 15.7‒16.7). The 5th and the 95th percentiles were estimated as 10.4 (95% CI: 9.8‒11.0) and 25.3 days (95% CI: 23.9‒27.0) (Table 2). The recovery time increased significantly with age (p < 0.01) and was substantially shorter after 22 January 2020 (14.1; 95% CI: 13.6‒14.6) than before (18.3 days; 95% CI: 17.7‒18.9). The epidemic phases were defined for the analysis of recovery times using a cut-off date of 22 January rather than 25 January to ensure an adequate number of observations in both phases. We found no substantial sex or geographical difference, although patients from Hubei province had slightly longer recovery times (Table 2).

## Discussion

Using publicly accessible data on COVID-19 patients and transmission clusters, we conducted a systematic inference on key epidemiological parameters of disease and transmission characteristics. These estimates are needed to inform public health control policies such as determining the duration of quarantine of close contacts and epidemic modelling efforts such as forecasting the timing of the peak of the epidemic [20]. The literature on the distributions of the incubation period and serial interval has been growing, yet most studies during the early pandemic were limited to either a small sample size (< 300) [13,14,24-29], a local region [30-32] or a particular demographic group [33]. Several studies had a sample size comparable to that in our study for estimating the incubation period, but the exposure period was either set to the departure time from Wuhan or constrained to be 3 days or shorter [34-36], which may have introduced bias. We found a single study that evaluated the generation interval of COVID-19 in two cities, Singapore and Tianjin, using a rigorous statistical model [37]. With a larger sample size and a broader geographical coverage, our study contributes valuable additional information about this important epidemiological aspect of COVID-19.

Our estimate for the median incubation period, 7.2 days as at 11 February 2020, is longer than most estimates for severe acute respiratory syndrome (SARS) (4‒7 days) and Middle-East respiratory syndrome (MERS) (4.5‒6 days) [38,39]. Our estimate is also longer than the majority of estimates for COVID-19 (mean: 4.8‒6.5 days; median: 3.0‒6.1 days) [1,12-17,24-28] but is similar to two large studies (mean or median: 7 days) [34,36] during the early pandemic. Estimates of ca 8 days for the mean or median incubation period were reported in two studies, one of which focused on cases in Beijing, China [32,35]. Some of these recent estimates were based on fewer cases from the early phase of the outbreak and are actually similar to our estimate for the same phase, ca 4.4 days before 25 January 2020 [14,16]. The estimated median of 6.5 days during the middle phase, 25‒31 January 2020, is similar to the mean of 6.4 days estimated by another study on patients outside Wuhan of China during 20–28 January 2020 [13]. This increasing trend became more dramatic in the later phase, reaching a median of 11.5 days. Two factors might have contributed to this trend: As the outbreak unfolded, mild cases or asymptomatic infections were probably more frequently detected as a result of improved diagnosis, more active contact tracing and increasing laboratory testing capacity. Historically, mild infections with SARS-CoV were associated with a longer incubation period [40]. The longer incubation period among paediatric cases and the fact that most paediatric patients tended to be mild also support the possibility that mild cases may have longer incubation periods [41]. However, while the proportion of children indeed increased with time in our dataset, the overall proportion of children is lower than 10% and thus unlikely to fully account for the observed variation of the incubation period over time. Another potential factor likely to affect the estimated duration of the incubation period are changes in epidemiological investigations, e.g. asking about a longer history back in time for contact tracing.

The serial interval has long been used as a surrogate for the generation interval, as the latter cannot be observed directly. The estimated medians were similar, 4.7 days for the serial interval and 4.6 days for the generation interval. The 5th and 95th percentiles of the serial interval were more extreme than those of the generation interval, indicating more variation in the former. Both the similarity in the median and the difference in variation are expected, given that the distribution of the incubation period does not change the mean but adds more variability when we use the serial interval to approximate the generation interval. Previous and recent studies reported a wide range of mean or median durations of serial intervals, from 4 to 7.5 days [1,27,42-47]. The majority of these estimates fall in the range of 4–5 days, close to the median of 4.7 days we estimated [27,42-46]. Two studies had sample sizes comparable to our study [44,46], but one study excluded all negative values of observed serial intervals [46]. The other study estimated a slightly shorter mean of 3.96 days based on 468 possible transmission pairs [44]. The median serial interval we estimated for COVID-19 is much shorter than the estimated means for SARS (7‒11 days) and MERS (12.6‒14.6 days) [9,10,12,38,45,48,49], indicating faster human-to-human transmission of the new coronavirus. In contrast to the temporal trend of the incubation period, the median serial interval decreased over time from 5.9 days before 25 January 2020 to 3.4 days after, and a similar but less dramatic contraction was seen for the generation interval. This trend is similar to what was observed for SARS in Singapore where the mean serial interval decreased from 10 days to less than 8 days after control measures were introduced [48]. The contraction of serial and generation interval over time could be explained partially by faster detection and isolation of cases and their close contacts during the later epidemic phase, leaving fewer days for transmission to occur.

Our estimate for the mean generation interval, 4.6 days, is in line with previous estimates of 5.2 days for Singapore and 3.95 days for Tianjin, China [37]. We estimated that the infectivity peaked 1 day before symptom onset on average and that ca 70% of transmission occurred before symptom onset. The proportion of pre-onset transmission based on our study is higher than the estimate for Singapore (48%) but similar to Tianjin (62%) [37]. The possibility of transmission during the incubation period has been indicated by a workplace cluster [50]. Transmission from asymptomatic infections has also been observed both inside and outside China [50,51], and some of these asymptomatic infections might have developed symptoms later assuming the possibility of a long incubation period. The role that asymptomatic carriers of SARS-CoV-2 have played in this epidemic is not clear, but such a high proportion of transmissions during the incubation period is alarming because the estimation was based on transmission pairs with symptomatic secondary cases. This finding could indicate that extensive contact tracing and diagnostic tools are important during the incubation period.

The estimated median of 16.2 days from symptom onset to hospital discharge among COVID-19 patients was comparable to that for MERS (12‒20 days) but shorter than for SARS (29.7 days) [18,39,52‒54]. Our estimate is shorter than two recent estimates (mean durations of 19.4 and 24.7 days) for COVID-19 [55,56]. Our study indicated that the recovery of paediatric cases could be faster than of elderly patients, probably because they have mild symptoms, but this observation needs to be confirmed by future studies with more paediatric cases [20]. The shortened disease course of recovered patients during the later epidemic phase (after 22 January 2020) is likely to reflect improvement in clinical care as healthcare givers became more experienced with the disease.

This study was subject to several limitations. Recall bias is inherent in self-reported exposure data, and we had to make subjective adjustment when details were lacking or conflicting, e.g. when the exposure intervals of the primary and secondary cases were occasionally reported as the same date, we assumed 0.5 days for the generation interval. Similarly, some of the COVID-19 patients included in the estimate of time from symptom onset to discharge were not available for the estimates of incubation period and serial/generation interval because their exposure information was missing. In addition, secondary cases could have been exposed to infectious sources other than the primary case, leading to contraction in the generation interval, i.e. shorter than it would have been had there been only one infectious source. Furthermore, mild cases might have a different disease profile compared with severe cases, but lack of the information on disease severity in our data prevented us from exploring such heterogeneity. Given the overall underdetection of mild cases, our estimates may be biased towards severe cases. Finally, cases and transmission pairs from Hubei Province and particularly Wuhan, the epicentre of the pandemic, were severely under-represented in our database because publicly available data from that region are scarce, which could have introduced bias into our estimates. 

## Conclusion

The long incubation period and short generation interval of COVID-19 have made it challenging to control the epidemic. The current standard quarantine period in China is 14 days, but according to our estimation, at least 5% of incubation periods could be longer than that. Public health authorities may consider a slight extension of isolation to, for instance 19 days, to reduce this proportion to less than 1%. The short generation interval and the high infectivity during the incubation period implies the necessity of extensive and efficient contact tracing, e.g. to screen contacts of contacts regardless of their symptom status, and aggressive adoption of prevention measures such as physical distancing and wearing face masks in crowded places. We recommend continuous collection of exposure history, especially within clusters of cases, with more details using nationally standardised forms to facilitate timely and accurate assessment of the key epidemiological parameters and transmissibility of the disease, which will in turn offer the necessary input for epidemic forecasting, intervention evaluation and public health decision making.

## Supplementary Data

752000 Supplement
