# Supplementary material for: Epidemiological parameters of COVID-19 and its implication for infectivity among patients in China, 1 January to 11 February 2020
Source: Euro Surveill. 2020 Oct 8;25(40):2000250. doi: 10.2807/1560-7917.ES.2020.25.40.2000250 (PMC7545819; doi:10.2807/1560-7917.ES.2020.25.40.2000250)

Supplementary Material for:

Natural history of disease of the novel coronavirus and its implication for infectivity among patients in China

This supplementary material is hosted by Eurosurveillance as supporting information alongside the article [Natural history of disease of the novel coronavirus and its implication for infectivity among patients in China], on behalf of the authors, who remain responsible for the accuracy and appropriateness of the content. The same standards for ethics, copyright, attributions and permissions as for the article apply. Supplements are not edited by Eurosurveillance and the journal is not responsible for the maintenance of any links or email addresses provided therein.

1. Supplementary Figure S1
2. Supplementary Figure S2
3. Supplementary Table S1
4. Supplementary Figure S3

Figure S1. The source and reproduced websites for publishing transmission pairs of the COVID-19 cases in our database used in the estimations of generation and serial intervals. (A) Pies represent the proportion of the type of websites; (B) Bars indicate the frequency of the number of websites publishing these transmission pairs.

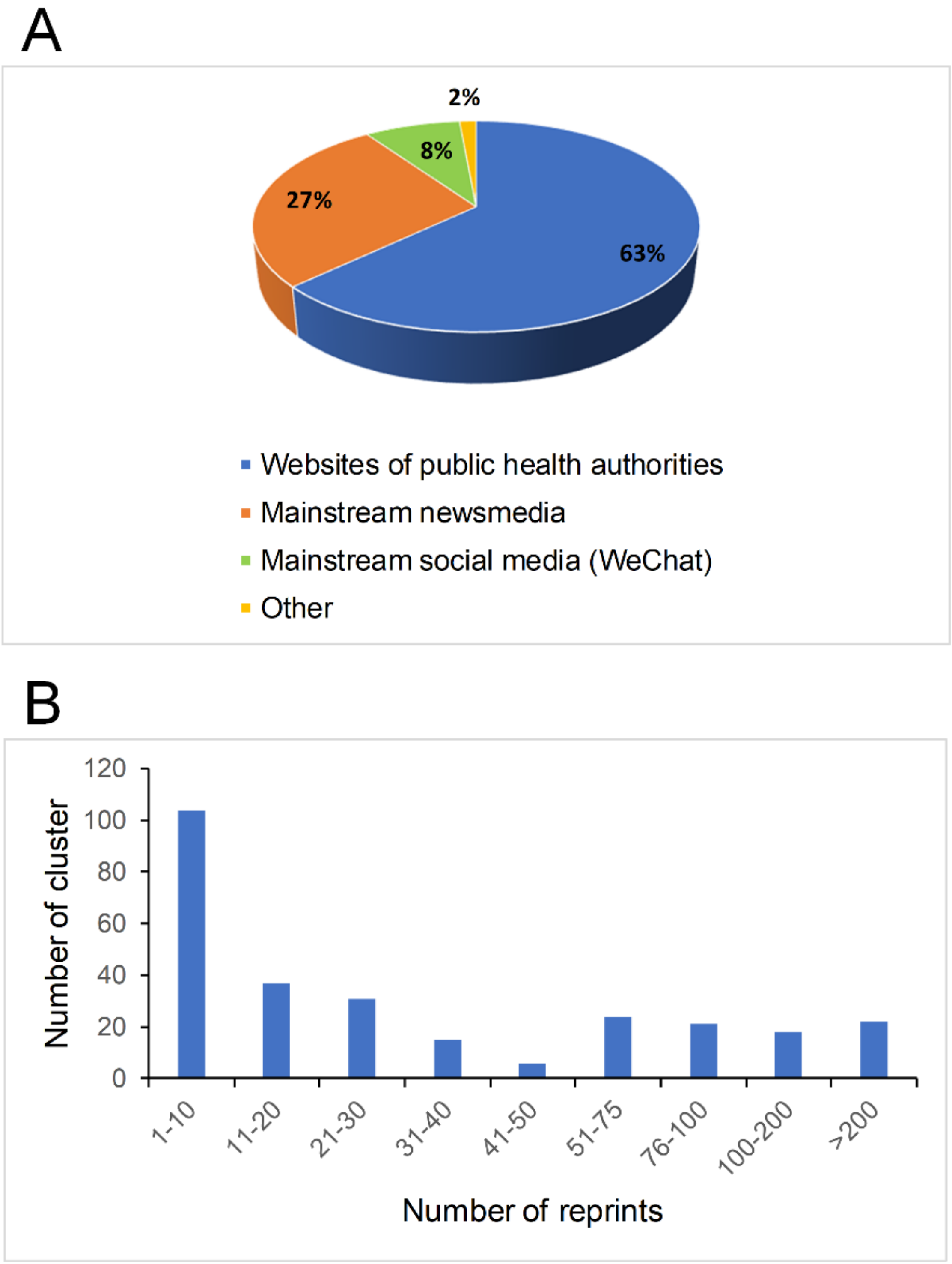

Figure S2. The temporal curves of COVID-19 cases in our database, compared with the overall number of cases in China (A), Guangdong Province (B), and Henan Province (C) from 1 January 2020 and 11 February 2020. The blue (left Y axis) and grey (right Y axis) bars represent the number of the COVID-19 cases in our database and overall number of cases reported in China and Guangdong or Henan provinces, respectively.

A

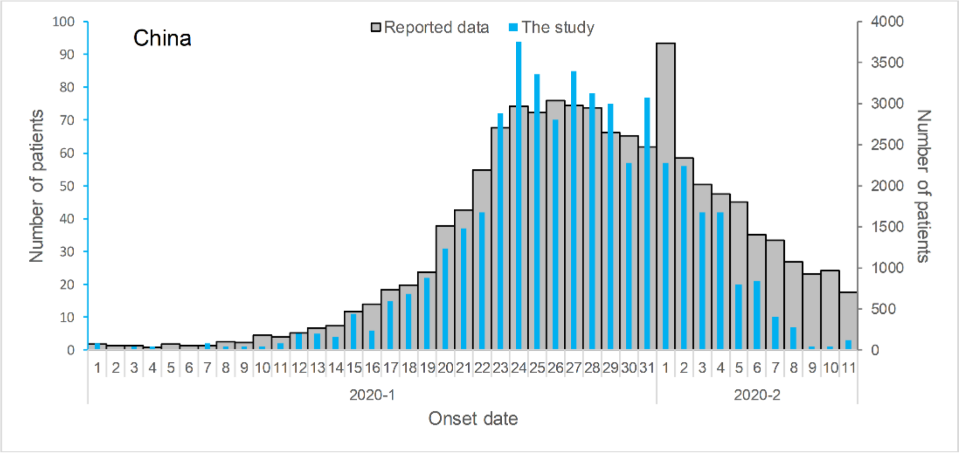

B

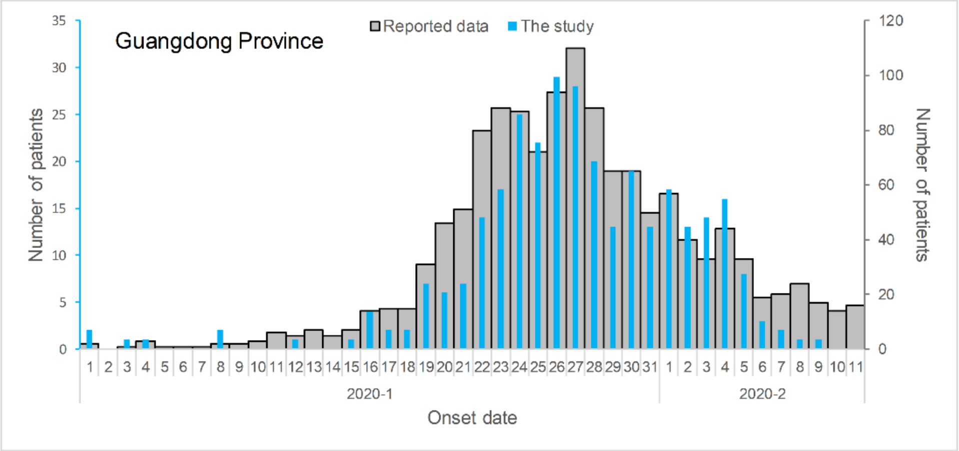

C

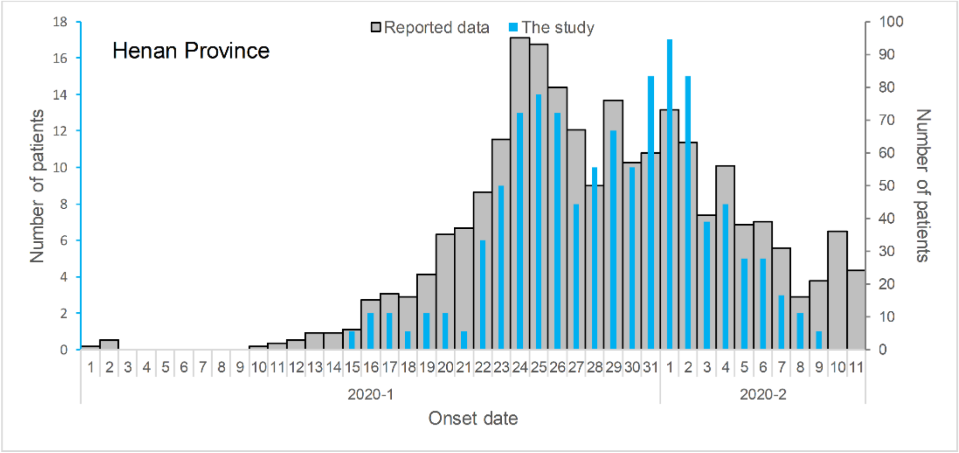

Table S1. Demographic characteristics of the patients found by internet search and used for estimating distributions of the incubation period, serial interval, generation interval and Time from symptom onset to discharge. Patients used for estimating generation interval is a subset of, and their demographics are similar to, those used for estimating serial interval.

Characteristic	Incubation period (n=1158)	Serial interval* (n=265)	Time from symptom onset to discharge (n=455)
Age, years, median (IQR)	43 (33–56)	46 (36–57)	39 (31–50)
<18	61 (5.3）	0 (0)	16 (3.5）
18–45	500 (43.2）	110 (41.5）	228 (50.2）
45–60	334 (28.8）	81 (30.6）	117 (25.7）
≥60	193 (16.7）	50 (18.9）	50 (11.0）
Unknown	70 (6.0）	24 (9.1）	44 (9.7）
Sex, n (%)			
Male	587 (50.7）	172 (64.9)	225 (49.6)
Female	568 (49.1）	86 (32.5)	205 (45.0)
Unknown	3 (0.3）	7 (2.6)	25 (5.5)
Location, n (%)			
North	518 (44.7）	140 (52.8)	88 (19.3)
South	640 (55.3）	125 (47.2)	367 (80.7)
Period, n (%)			
Before Jan 25, 2020	278 (24.0）	153 (57.7）	369 (81.1）
Jan 25–Jan 31, 2020	543 (46.9）	94 (35.5）	85 (18.7）
After Jan 31, 2020	337 (29.1）	18 (6.8）	1 (0.2）

Note: *represents for the index cases.

Figure S3. Estimated distributions of the time from symptom onset to hospital discharge based on public data on COVID-19 cases in China as of 11 February 2020. Estimates are shown for overall (A) and for epidemic phases, defined as symptom onsets before 22 January 2020 (B) and during 22 January–11 February 2020 (C). Parametric models fitted are Weibull (green dashed), gamma (blue dashed), log-normal (purple dashed) and log-logistic (red solid). Density of observed distribution is shown as the histogram. Vertical lines indicate mean (dark blue) and median (orange) of the log-logistic distribution.

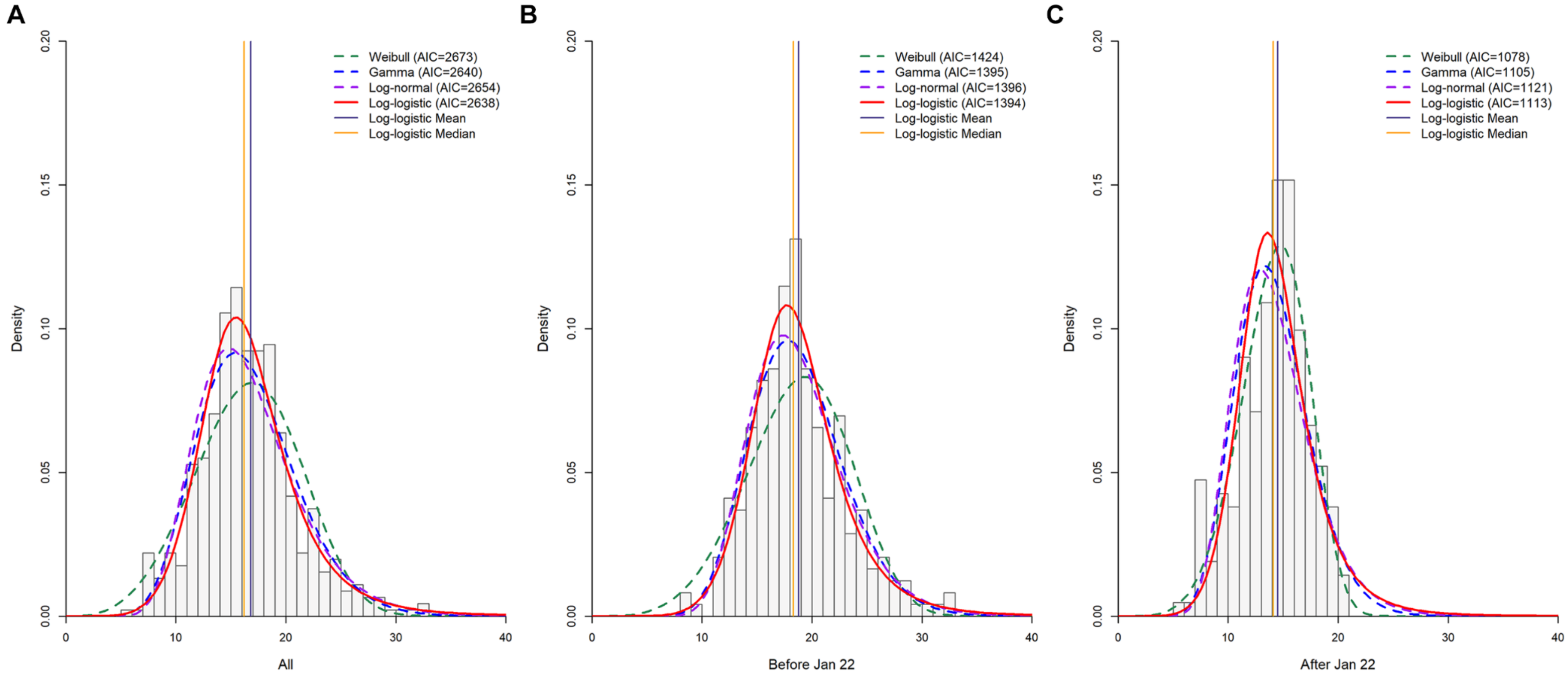

Supplement: Supplement [file 20-00250_FANG_Supplementary_Material.pdf]
